# Adenosine signaling in tumor immune escape: metabolic checkpoints, myeloid suppression, and combination immunotherapy

**DOI:** 10.3389/fonc.2026.1915472

**Published:** 2026-08-24

**Authors:** Rui Li, Shengbiao Li, Tongtong Zhang

**Affiliations:** 1 School of Basic Medical Sciences, Southwest Medical University, Luzhou, China; 2 Obesity and Metabolism Medicine-Engineering Integration Laboratory, Department of General Surgery, Affiliated Hospital of Southwest Jiaotong University, The Third People’s Hospital of Chengdu, Chengdu, China

**Keywords:** adenosine, CD73/CD39, immunotherapy resistance, myeloid suppression, tumor microenvironment

## Abstract

Adenosine is a central metabolic regulator of tumor immune escape, shaping the tumor microenvironment (TME) through suppression of effector lymphocytes and reprogramming of myeloid populations. Extracellular adenosine is generated primarily via the ectonucleotidases CD39 and CD73 and signals through A2A (A2AR) and A2B (A2BR) receptors on T cells, NK cells, tumor-associated macrophages (TAMs), myeloid-derived suppressor cells (MDSCs), and dendritic cells. This pathway promotes T-cell exhaustion, inhibits cytotoxicity, and enhances myeloid-driven immunosuppression, creating metabolic and spatial barriers that limit the efficacy of immune checkpoint blockade, STING agonists, radiotherapy, and emerging photothermal or nanomaterial-based therapies. Preclinical studies demonstrate that targeting the adenosine axis—via CD39/CD73 inhibition, receptor blockade, or combination strategies—can restore immune effector function, reprogram suppressive myeloid niches, and potentiate antitumor immunity. Spatial and circulating biomarkers, including tumor and exosomal CD73, adenosine gradients, and TAM/MDSC infiltration, may guide patient stratification and optimize combinatorial immunotherapy. Integrating adenosine-targeted approaches with PD-1/PD-L1 blockade, STING agonists, or adoptive cell therapies offers a rational strategy to overcome resistance and improve therapeutic outcomes. This review summarizes recent experimental evidence on the mechanisms of adenosine-mediated immune suppression, highlights translational opportunities, and discusses strategies for personalized and combination therapies aimed at dismantling metabolic immune checkpoints in cancer.

## Highlights

- Adenosine suppresses T/NK cells and remodels myeloid cells, driving tumor immune escape.
- CD39/CD73–A2AR/A2BR axis mediates resistance to checkpoint blockade, STING, and radiotherapy.
- Combination therapy and biomarker-guided adenosine targeting may enhance immunotherapy efficacy.

## 1 Introduction

Tumor immune evasion remains one of the primary obstacles limiting the efficacy of cancer immunotherapy (1–5). While immune checkpoint inhibitors (ICIs) targeting PD-1/PD-L1 and CTLA-4 have transformed the therapeutic landscape in multiple malignancies, a significant proportion of patients fail to respond, and among initial responders, acquired resistance frequently develops (6–10). Traditional paradigms of immune escape have largely focused on the upregulation of inhibitory checkpoints or the induction of T cell exhaustion. However, emerging evidence highlights the pivotal role of metabolic reprogramming within the tumor microenvironment (TME) in driving immunosuppression, which operates independently or synergistically with classical checkpoint pathways.

Among these metabolic mechanisms, extracellular adenosine (eADO) and its enzymatic machinery—particularly the ectonucleotidases CD39 and CD73, which sequentially hydrolyze extracellular ATP to adenosine—have emerged as critical regulators of tumor-mediated immune suppression (11–15). Adenosine accumulates in hypoxic or metabolically stressed regions of the TME, where it engages A2A (A2AR) and A2B (A2BR) receptors expressed on both lymphoid and myeloid immune cells (16–20). Engagement of these receptors inhibits cytotoxic T lymphocyte (CTL) and natural killer (NK) cell functions by suppressing proliferation, cytokine secretion (e.g., IFN-γ and TNF-α), and granule-mediated cytotoxicity, effectively creating an immune-privileged niche for tumor cells (21). Importantly, the adenosine axis also exerts profound effects on myeloid populations, including tumor-associated macrophages (TAMs) and myeloid-derived suppressor cells (MDSCs), which are recruited and functionally polarized by adenosine signaling. This not only amplifies the immunosuppressive milieu but also establishes feed-forward loops, wherein TAMs and MDSCs further enhance adenosine production, stabilize hypoxic niches, and suppress antigen presentation. The resulting myeloid-mediated immune suppression contributes to T cell dysfunction and supports tumor progression, highlighting the dual role of adenosine in regulating both lymphoid and myeloid compartments (22).

Recent experimental evidence underscores the clinical and translational relevance of this pathway. For instance, elevated expression of CD73 on tumor cells or in circulating exosomes has been correlated with poor responses to PD-1 blockade, providing a predictive biomarker for immunotherapy resistance (21). Additionally, preclinical studies have demonstrated that pharmacological or genetic blockade of CD39/CD73 or antagonism of A2AR/A2BR can restore effector T cell and NK cell function, reprogram suppressive myeloid populations, and enhance responses to ICIs or other immunomodulatory therapies (23). The concept of adenosine signaling as a “metabolic immune checkpoint” has therefore gained significant traction. Unlike classical checkpoints, this axis integrates metabolic cues from the TME with immune cell regulation, linking tissue hypoxia, nutrient deprivation, and extracellular nucleotide accumulation to functional suppression of antitumor immunity. Understanding these interactions is crucial for the rational design of combination therapies, such as co-targeting adenosine pathways with PD-1/PD-L1 inhibitors, STING agonists, radiotherapy, or adoptive cell therapies, to overcome intrinsic and acquired resistance mechanisms.

In this review, we aim to provide a comprehensive summary of the latest experimental findings onward on adenosine-mediated immune suppression in cancer. We will highlight the dual impact of adenosine on lymphoid and myeloid populations, explore its mechanistic underpinnings, and discuss translational strategies for targeting this pathway to enhance the efficacy of current and emerging immunotherapies. By integrating mechanistic insights with preclinical and translational data, this review seeks to establish a framework for understanding how metabolic checkpoints contribute to tumor immune escape and guide the development of next-generation immunotherapeutic interventions.

## 2 Adenosine production in the tumor microenvironment

### 2.1 Ectonucleotidases CD39/CD73

The accumulation of extracellular adenosine (eADO) in the tumor microenvironment (TME) is primarily mediated by the sequential enzymatic activity of ectonucleotidases CD39 (ENTPD1) and CD73 (NT5E). CD39 hydrolyzes extracellular ATP and ADP to AMP, which is then converted to adenosine by CD73 (24–29). This pathway is particularly relevant in tumors, where cellular stress, necrosis, and immune cell activation release large amounts of ATP into the extracellular space, providing abundant substrate for adenosine production. Both tumor cells and various stromal and immune populations express CD39 and CD73, including tumor-associated macrophages (TAMs), myeloid-derived suppressor cells (MDSCs), and dendritic cells, creating a robust immunosuppressive niche (30–33). Importantly, adenosine generated by these enzymes exerts potent inhibitory effects on effector T and NK cells, limiting their proliferation, cytokine secretion, and cytotoxic activity.

Recent studies have highlighted the significance of exosome-mediated CD73 as a mechanism of systemic immune suppression (34–38). Tumor-derived exosomes enriched with CD73 can generate adenosine in circulation, dampening T cell activity at distant sites. Notably, elevated levels of exosomal CD73 in the serum have been associated with poor response to anti-PD-1 therapy, indicating its potential as a predictive biomarker for immunotherapy outcomes (21). Beyond tumor cells, intrinsic alterations in the TME can further augment CD73 expression on myeloid cells. For example, MET amplification in tumors has been shown to upregulate CD73 on TAMs, suppressing STING-mediated type I interferon responses and limiting T cell recruitment (39). Similarly, senescent tumor cells can induce CD73 expression on surrounding TAMs, creating an adenosine-rich environment that reinforces immune evasion (40). These findings underscore the multifaceted contribution of CD39/CD73 to both local and systemic immunosuppression.

### 2.2 Spatial and metabolic regulation

The production and activity of CD39/CD73 are tightly regulated by the metabolic and spatial context of the TME. Hypoxia, a hallmark of many solid tumors, stabilizes HIF-1α, which transcriptionally upregulates CD73, enhancing adenosine generation (41–43). In addition, metabolic byproducts such as lactate, generated from aerobic glycolysis, can further induce CD39/CD73 expression on both tumor and stromal cells, reinforcing local immunosuppression (44). Thus, extracellular adenosine in the TME is mainly generated through the coordinated enzymatic activity of CD39 and CD73. CD39 hydrolyzes extracellular ATP and ADP into AMP, whereas CD73 further converts AMP into adenosine. This process is closely coupled to hypoxia and lactate metabolism. Hypoxia promotes adenosine accumulation by stabilizing HIF-1α and increasing CD73 expression, while lactate-rich and acidic metabolic niches can further enhance CD39/CD73 expression on tumor, stromal, and myeloid cells (34, 38). Conversely, increased adenosine signaling can reinforce hypoxic and lactate-enriched immunosuppressive niches by suppressing effector T/NK-cell activity, promoting TAM/MDSC-mediated immune suppression, and supporting tumor metabolic adaptation. Therefore, hypoxia, lactate metabolism, and CD39/CD73-mediated adenosine production form a feed-forward immunometabolic circuit that amplifies immune escape within the TME.

Spatial heterogeneity of adenosine within the tumor also plays a critical role in shaping immune responses. Regions with high adenosine concentrations often coincide with hypoxic or metabolically stressed niches, where effector T cells are excluded or functionally suppressed. Mapping studies have demonstrated that these adenosine-rich microdomains create gradients of immune inhibition, limiting T and NK cell infiltration into tumor cores and promoting immune escape (45). Functionally, an adenosine gradient creates a spatially layered immune-suppressive barrier. In peripheral or less hypoxic tumor regions, T cells may still enter the tumor bed and retain partial effector activity. However, as T cells migrate toward tumor cores or hypoxic, CD39/CD73-rich niches, progressively higher extracellular adenosine concentrations can activate A2AR on T cells and suppress TCR signaling, cytokine production, proliferation, and cytotoxic activity (46). This produces a “functional exclusion” state in which T cells may be present at the invasive margin or stromal interface but fail to penetrate or remain active within adenosine-rich tumor nests. In parallel, adenosine-rich regions often overlap with TAM/MDSC-enriched niches, where A2BR-mediated myeloid suppression further reduces antigen presentation, promotes inhibitory cytokine production, and reinforces T-cell dysfunction. Therefore, the spatial distribution of adenosine influences not only the number of infiltrating T cells, but also their location, activation state, and capacity to execute tumor-cell killing.

**Figure 1:**
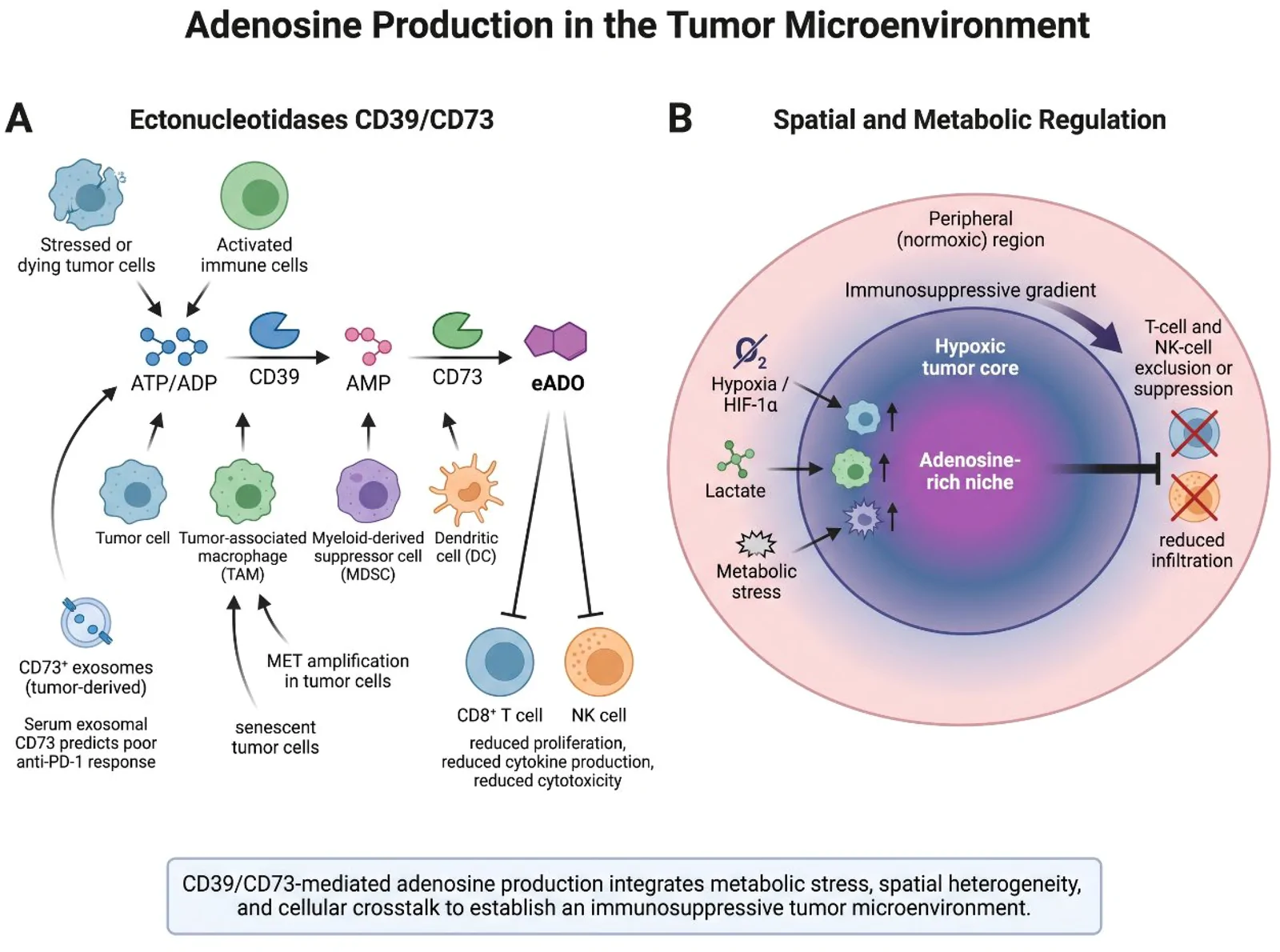
Mechanistic overview of adenosine production in the tumor microenvironment. **(A)** Ectonucleotidases CD39/CD73 sequentially convert extracellular ATP/ADP released by stressed tumor and immune cells into AMP and adenosine. Tumor cells, TAMS, MDSCs, DCs, and exosomes contribute to adenosine accumulation, which suppresses CD8? T-cell and NK-cell activity. MET amplification and tumor senescence further enhance TAM CD73 expression. **(B)** Spatial and metabolic regulation: hypoxia and lactate reinforce CD39/CD73 expression, generating spatial adenosine gradients that restrict immune infiltration.

Together, these studies indicate that CD39/CD73-mediated adenosine production is not merely a biochemical pathway, but a central regulatory mechanism integrating metabolic cues, spatial constraints, and cellular crosstalk to modulate antitumor immunity. Targeting this pathway has the potential to reshape the TME from an immunosuppressive to an immune-permissive state, providing a rational strategy for enhancing the efficacy of existing immunotherapies. **Figure 1** illustrates how CD39/CD73 enzymatic activity integrates metabolic cues, spatial heterogeneity, and myeloid cell regulation to create an immunosuppressive TME. It highlights the main sources, regulatory signals, and inhibitory impact on effector lymphocytes.

## 3 Adenosine signaling pathways

Adenosine generated in the tumor microenvironment exerts its immunosuppressive effects primarily through engagement of G protein-coupled adenosine receptors, notably A2A (A2AR) and A2B (A2BR). These receptors are differentially expressed on lymphoid and myeloid populations, mediating distinct yet overlapping inhibitory effects on antitumor immunity. In addition, emerging studies indicate that adenosine can influence tumor cells and stromal components through noncanonical pathways, affecting metabolic adaptation, signaling feedback, and therapeutic resistance.

### 3.1 A2A receptor

The A2AR is highly expressed on effector T cells and natural killer (NK) cells, where it functions as a critical metabolic immune checkpoint (47–51). Engagement of A2AR by extracellular adenosine activates adenylate cyclase, elevating intracellular cAMP levels and subsequently inhibiting T cell receptor (TCR) signaling and downstream cytotoxic programs. This leads to diminished proliferation, impaired cytokine secretion (including IFN-γ and TNF-α), and reduced granzyme- and perforin-mediated cytotoxicity, effectively blunting antitumor immune responses (52, 53). Experimental blockade of A2AR has been shown to restore effector functions of cytotoxic T lymphocytes (CTLs) and NK cells. Preclinical models demonstrate that A2AR antagonism enhances T cell proliferation, IFN-γ secretion, and perforin-mediated killing, resulting in improved tumor control. Furthermore, when combined with PD-1 immune checkpoint blockade, A2AR inhibition synergistically amplifies antitumor efficacy, suggesting that adenosine-A2AR signaling is a key barrier to ICI responsiveness in multiple tumor models (53). These findings highlight A2AR as a promising target for enhancing both adaptive and innate immune-mediated tumor clearance. Functionally, A2AR should be regarded as the dominant lymphoid inhibitory receptor within the adenosine axis. In CD8+ T cells, A2AR activation mainly suppresses proximal TCR signaling and downstream effector programs, leading to reduced clonal expansion, decreased IL-2, IFN-γ, and TNF-α production, impaired granzyme B/perforin-mediated cytotoxicity, and weakened tumor-cell killing (18). In NK cells, A2AR signaling similarly diminishes activating-receptor-driven cytotoxicity, cytokine secretion, degranulation, and immune surveillance. Therefore, A2AR-mediated suppression is most evident in tumors where T cells or NK cells are present but functionally restrained by high extracellular adenosine. In this setting, A2AR blockade primarily aims to release lymphoid effector cells from metabolic inhibition and restore their cytotoxic activity.

### 3.2 A2B receptor

In contrast to A2AR, the A2BR is predominantly expressed on myeloid populations, including tumor-associated macrophages (TAMs) and myeloid-derived suppressor cells (MDSCs). Adenosine engagement of A2BR activates intracellular signaling pathways that enhance the immunosuppressive phenotype of these cells, including upregulation of ARG1, IL-10, TGF-β, and PD-L1 (54–59). Consequently, TAMs and MDSCs inhibit CTL function and limit NK cell cytotoxicity, contributing to an immunosuppressive TME and promoting tumor progression (23). Pharmacologic inhibition of A2BR has been demonstrated to reverse these suppressive effects, restoring T cell proliferation and cytokine production, while reducing the suppressive capacity of TAMs and MDSCs. Preclinical studies indicate that targeting A2BR in combination with checkpoint inhibitors enhances antitumor immunity and overcomes resistance mechanisms mediated by myeloid cells (22). Thus, while A2AR primarily regulates lymphoid effector cell function, A2BR serves as a critical mediator of myeloid-driven immunosuppression, emphasizing the importance of receptor-specific strategies in therapeutic design. By contrast, A2BR is more closely associated with myeloid and stromal reprogramming than with direct lymphoid inhibition. In TAMs, A2BR activation promotes an immunoregulatory macrophage phenotype characterized by increased IL-10, TGF-β, ARG1, VEGF, and PD-L1 expression, reduced antigen-presenting capacity, and enhanced tissue-remodeling or angiogenic activity. These changes indirectly suppress T-cell activation and infiltration by creating a macrophage-dominant immune-excluded niche. In MDSCs, A2BR signaling stabilizes suppressive activity by enhancing arginine metabolism, inhibitory cytokine production, oxidative stress-related suppression, and PD-L1-associated checkpoint activity (60). As a result, A2BR blockade is expected to weaken myeloid-mediated immune resistance, improve antigen presentation, reduce suppressive cytokine networks, and facilitate CD8+ T-cell infiltration into tumor tissue. Thus, A2AR and A2BR mediate complementary but nonidentical layers of adenosine-driven immune suppression. A2AR mainly functions as a direct inhibitory checkpoint on lymphoid effector cells, limiting T-cell and NK-cell activation, cytokine production, and cytotoxic killing. A2BR mainly acts as a myeloid- and stroma-associated remodeling receptor, promoting TAM/MDSC suppressive polarization, impaired antigen presentation, angiogenic and tissue-remodeling programs, and immune exclusion. This distinction has therapeutic implications: A2AR antagonism may be most useful for restoring pre-existing but metabolically inhibited T/NK-cell responses, whereas A2BR blockade may be particularly valuable in myeloid-rich, hypoxic, or immune-excluded tumors where suppressive TAMs and MDSCs dominate the resistance landscape.

### 3.3 Noncanonical functions of adenosine signaling

Beyond its canonical role in immune suppression, adenosine signaling exhibits noncanonical functions that influence tumor cell metabolism and therapeutic resistance. For instance, CD73 not only generates adenosine but also contributes to tumor metabolic adaptation (61–65). CD73 expression promotes oxidative phosphorylation and glycolytic flexibility, enhancing tumor cell survival under metabolic stress and conferring resistance to chemotherapeutic and targeted therapies (66). Furthermore, adenosine signaling can negatively regulate innate immune sensing pathways, such as the STING pathway, which is critical for type I interferon production and antitumor immunity. Elevated adenosine levels in the TME have been shown to attenuate STING-mediated signaling, dampening dendritic cell activation and T cell priming, and thereby limiting responses to immunotherapies including STING agonists and checkpoint inhibitors (67). These observations indicate that adenosine functions not only as a metabolic checkpoint on immune cells but also as a modulator of tumor-stroma signaling, creating feedback loops that reinforce immune evasion and therapy resistance.

**Figure 2:**
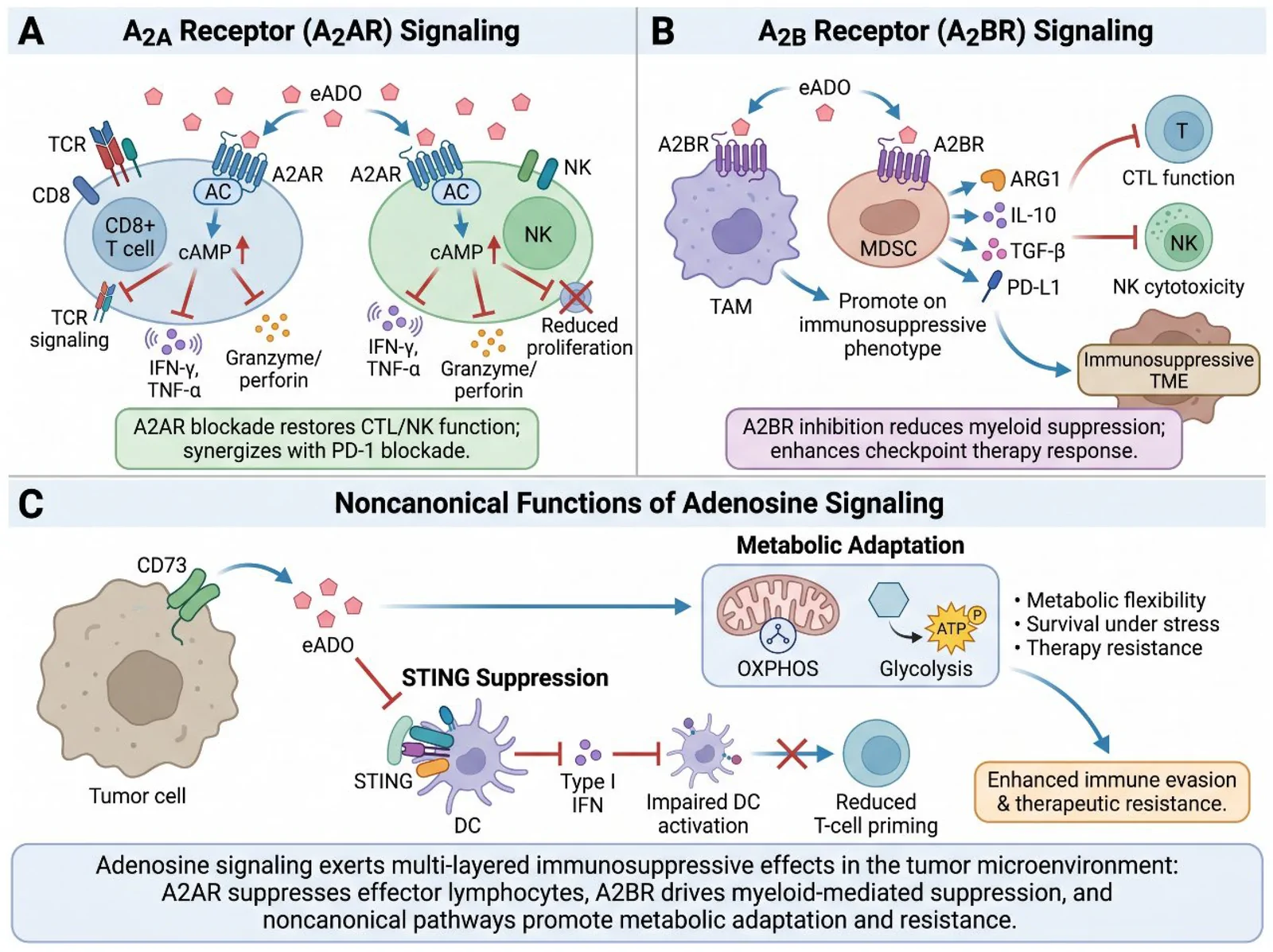
Mechanistic overview of adenosine signaling pathways in tumor immune escape **(A)** A2A receptor signaling in CD8+T cells and NK cells leads to cAMP-dependent immunosuppression, which can be restored by A2AR blockade. **(B)** A2B receptor signaling in TAMs and MDSCs promotes immunosuppressive mediators, including ARG1, IL-10, TGF-β, and PD-L1. **(C)** Noncanonical functions of adenosine signaling, including tumor metabolic adaptation and STING pathway suppression, resulting in impaired dendritic cell function and enhanced immune evasion and therapeutic resistance.

The adenosine receptor axis exerts a multi-layered immunosuppressive effect in the TME. A2AR predominantly inhibits effector lymphocytes, A2BR mediates myeloid-driven suppression, and noncanonical pathways involving tumor metabolic adaptation and STING modulation further enhance immune escape. Together, these mechanisms underscore the central role of adenosine signaling in limiting antitumor immunity and highlight the potential of receptor-specific or combinatorial targeting strategies to overcome tumor immune evasion. **Figure 2** illustrates the major signaling routes through which adenosine suppresses antitumor immunity in the tumor microenvironment. It highlights the distinct roles of A2AR in lymphoid cells, A2BR in myeloid cells, and noncanonical pathways linking adenosine signaling to metabolic adaptation and resistance. **Table 1** lists the main molecular targets within the adenosine pathway, their primary cellular localization, and the resulting functional effects on immune or tumor cells. It provides a concise reference for understanding how adenosine regulates lymphoid and myeloid activity in the tumor microenvironment.

**Table 1:** Adenosine signaling targets and cellular effects.

Target/axis	Cell type	Functional effect	Therapeutic implication	References (PMID)
CD39	Tumor, TAM, MDSC	Converts ATP/ADP → AMP; source of adenosine	Enzyme inhibition can reduce eADO	(68)
CD73	Tumor, TAM, MDSC, DC, exosomes	Converts AMP → eADO; metabolic immune checkpoint	Blockade restores T/NK function; predictive biomarker	(69, 70)
A2AR	CD8+ T cells, NK cells	cAMP-mediated inhibition; reduces proliferation, cytokines, cytotoxicity	Antagonism restores effector function; synergizes with PD-1	(52, 53)
A2BR	TAMs, MDSCs	Enhances ARG1, IL-10, TGF-β, PD-L1; suppressive myeloid phenotype	Blockade reduces myeloid-mediated immunosuppression	(22, 23)

## 4 Myeloid suppression mediated by adenosine

Adenosine-mediated immunosuppression in the tumor microenvironment (TME) extends far beyond the direct inhibition of effector lymphocytes. Although cytotoxic T lymphocytes (CTLs) and natural killer (NK) cells are classical targets of extracellular adenosine, accumulating evidence indicates that myeloid populations are equally important mediators of adenosine-driven immune escape (71–74). Tumor-associated macrophages (TAMs), myeloid-derived suppressor cells (MDSCs), and dendritic cells (DCs) are not only responsive to adenosine receptor signaling but can also contribute to extracellular adenosine generation through the expression of CD39 and CD73. This creates a self-reinforcing suppressive network in which myeloid cells produce adenosine, respond to adenosine, and subsequently inhibit antitumor lymphocyte activity. Within the TME, adenosine-rich niches often overlap with hypoxic, metabolically stressed, and immune-excluded regions. These areas are frequently enriched with suppressive myeloid cells and depleted of functional CTLs. Through A2AR- and A2BR-dependent mechanisms, adenosine signaling promotes myeloid polarization toward immunoregulatory phenotypes, reduces antigen presentation, enhances suppressive cytokine production, and limits T-cell recruitment and activation. Therefore, the adenosine axis should be understood not only as a lymphocyte-intrinsic inhibitory pathway, but also as a central regulator of myeloid-cell programming and myeloid–lymphoid crosstalk. In this context, TAMs and MDSCs are not only passive targets of adenosine, but also active producers and amplifiers of adenosine-mediated suppression. By expressing CD39 and/or CD73, these myeloid populations convert extracellular ATP into adenosine, while A2AR/A2BR signaling further stabilizes their suppressive phenotype (75). This creates a feed-forward circuit in which adenosine-producing myeloid cells generate a metabolically hostile niche that limits T-cell priming, effector-cell infiltration, cytotoxic activity, and durable responses to immunotherapy.

### 4.1 Tumor-associated macrophages

Tumor-associated macrophages represent one of the most abundant and functionally plastic myeloid populations in solid tumors. Depending on local signals, TAMs can acquire either inflammatory, tumor-restraining phenotypes or immunosuppressive, tissue-remodeling phenotypes. In many established tumors, however, TAMs are skewed toward a suppressive state characterized by impaired antigen presentation, increased production of IL-10 and TGF-β, enhanced expression of immune checkpoint molecules, and promotion of angiogenesis and extracellular matrix remodeling. Adenosine signaling contributes substantially to this macrophage reprogramming. TAMs can express CD73, enabling them to generate extracellular adenosine locally (76–80). This TAM-derived adenosine suppresses CTL activation, reduces cytokine secretion, and promotes an immune-excluded TME. In addition, adenosine signaling can further reinforce suppressive macrophage polarization, creating a positive feedback loop in which TAMs both produce and respond to adenosine. This mechanism allows TAMs to function as active amplifiers of metabolic immune suppression rather than passive bystanders within the TME (40).

Environmental stressors such as tumor cell senescence, hypoxia, nutrient deprivation, and treatment-induced tissue damage may further enhance TAM-associated CD73 expression. Senescent tumor cells can secrete inflammatory and stromal-remodeling mediators that induce CD73 expression in surrounding macrophages, thereby increasing local adenosine production and strengthening immune evasion (81–85). Under hypoxic conditions, adenosine accumulation is further promoted by increased ectonucleotidase activity and impaired extracellular nucleotide clearance. These conditions favor the generation of adenosine-rich macrophage niches that suppress T-cell infiltration and cytotoxic function (40). Mechanistically, adenosine engagement of A2BR on TAMs can promote immunosuppressive macrophage programs, including increased expression of ARG1, IL-10, VEGF, and other mediators associated with tissue repair and tumor progression. These macrophage-derived factors impair T-cell proliferation, inhibit effector cytokine production, and support vascular remodeling, thereby creating a microenvironment that is poorly permissive to antitumor immunity. In this context, TAM-CD73 and A2BR signaling may contribute to primary or acquired resistance to immune checkpoint blockade by maintaining myeloid-dominant immune suppression even when PD-1/PD-L1 signaling is inhibited. Thus, targeting the adenosine pathway in TAMs may have multiple therapeutic effects: reducing extracellular adenosine accumulation, reprogramming macrophages toward a less suppressive phenotype, improving antigen presentation, and facilitating CTL infiltration into tumor tissue. These effects are particularly relevant for tumors characterized by macrophage-rich but T-cell-poor immune landscapes. At the functional level, adenosine reshapes TAMs from antigen-presenting or inflammatory macrophages toward an immunoregulatory, tissue-remodeling phenotype. A2BR signaling in TAMs increases the expression of ARG1, IL-10, TGF-β, VEGF, and PD-L1, while reducing effective antigen presentation and inflammatory cytokine production (81). These changes suppress CD8+ T-cell proliferation and cytotoxicity, impair NK-cell-mediated tumor surveillance, promote angiogenesis and matrix remodeling, and favor T-cell exclusion from tumor nests. TAM-derived CD73 further amplifies this process by increasing local adenosine production, thereby establishing a macrophage-centered suppressive niche. Thus, the adenosine pathway drives TAMs to function as both a source and an effector of immune suppression within the TME.

### 4.2 Myeloid-derived suppressor cells

MDSCs are heterogeneous immature myeloid cells that expand during cancer progression and potently suppress antitumor immunity (86–90). They are commonly divided into monocytic MDSCs and polymorphonuclear or granulocytic MDSCs, both of which can inhibit T-cell responses through distinct but overlapping mechanisms. These include arginine depletion, nitric oxide and reactive oxygen species production, secretion of suppressive cytokines, induction of regulatory T cells, and disruption of T-cell receptor signaling. The adenosine pathway provides an additional metabolic layer through which MDSCs exert immune suppression (91–95). Monocytic MDSCs can utilize the CD39/CD73–adenosine axis to generate extracellular adenosine and suppress effector T-cell activity. By converting extracellular ATP into adenosine, MDSCs shift the TME from an inflammatory ATP-rich environment toward an immunosuppressive adenosine-dominant state. This metabolic conversion is functionally important because extracellular ATP can promote immune activation through purinergic signaling, whereas adenosine suppresses T-cell proliferation, IFN-γ production, and cytotoxic function (96).

Adenosine also acts directly on MDSCs to stabilize and amplify their suppressive phenotype. Through A2AR and A2BR signaling, adenosine can enhance the expression of immunoregulatory mediators and increase the ability of MDSCs to inhibit T-cell responses. In this way, MDSCs participate in a feed-forward circuit: they generate adenosine through CD39/CD73 activity, respond to adenosine receptor signaling, and further suppress antitumor lymphocytes (97–101). This mechanism is particularly important in tumors with abundant myeloid infiltration, where checkpoint blockade alone may be insufficient to restore effective immunity. Mechanistically, adenosine strengthens the suppressive activity of MDSCs through both metabolic and receptor-mediated mechanisms. CD39/CD73-expressing MDSCs convert extracellular ATP, which normally supports inflammatory purinergic signaling, into adenosine, thereby shifting the local immune context from activation to suppression. In parallel, A2AR and A2BR signaling in MDSCs enhances suppressive programs such as arginine depletion, nitric oxide and reactive oxygen species production, IL-10 and TGF-β secretion, PD-L1 expression, and Treg-promoting activity (102–104). These effects inhibit T-cell receptor signaling, reduce IFN-γ production, limit cytotoxic lymphocyte expansion, and weaken antitumor effector responses. Therefore, adenosine-conditioned MDSCs help convert an inflamed or therapy-stressed tumor site into a metabolically suppressed microenvironment that remains resistant to immune checkpoint blockade.

Experimental blockade of the adenosine pathway can relieve MDSC-mediated suppression. Inhibition of CD39/CD73 enzymatic activity or antagonism of A2AR/A2BR signaling can restore T-cell proliferation, enhance IFN-γ secretion, and improve cytotoxic activity. Importantly, targeting adenosine signaling may also improve the responsiveness of tumors to checkpoint inhibitors and other immunotherapies by weakening the myeloid suppressive barrier (105). These findings support the concept that successful immunotherapy may require simultaneous reinvigoration of exhausted T cells and dismantling of MDSC-driven metabolic suppression. From a therapeutic perspective, MDSC-associated adenosine signaling may serve both as a biomarker and as a target. High infiltration of CD39/CD73-expressing MDSCs may identify tumors with strong metabolic immune suppression and poor responsiveness to PD-1/PD-L1 blockade. Conversely, combining adenosine-axis inhibitors with checkpoint blockade, radiotherapy, or myeloid-targeting agents may convert suppressive myeloid niches into more immune-permissive environments.

### 4.3 Dendritic cells

Dendritic cells are essential for initiating and sustaining adaptive antitumor immunity (106–109). By capturing tumor antigens, migrating to lymphoid structures, and presenting antigenic peptides to naïve or memory T cells, DCs bridge innate sensing and T-cell-mediated cytotoxicity. Effective DC maturation is characterized by increased expression of MHC molecules, co-stimulatory ligands such as CD80 and CD86, and inflammatory cytokines that support CTL priming. However, high levels of adenosine in the TME can compromise these functions and weaken the initiation of antitumor immune responses. Adenosine signaling suppresses DC maturation and antigen-presenting capacity (110–113). In adenosine-rich environments, DCs may exhibit reduced expression of co-stimulatory molecules, impaired production of proinflammatory cytokines, and diminished ability to prime functional CTLs. This limits the generation of effective antitumor T-cell responses and contributes to the formation of an immunologically “cold” TME (114). Such DC dysfunction is especially relevant in tumors where poor antigen presentation is a major determinant of resistance to immune checkpoint blockade. This DC-centered mechanism represents an important non-classical route by which adenosine weakens antitumor immunity (115, 116). Rather than only inhibiting cytotoxic lymphocytes after they have been activated, adenosine can act earlier in the cancer-immunity cycle by limiting antigen presentation and T-cell priming. Suppression of DC maturation reduces the expression of co-stimulatory molecules and inflammatory mediators required for productive CTL activation. Consequently, even when tumor antigens are released by spontaneous tumor-cell death, radiotherapy, chemotherapy, or STING agonists, adenosine-rich niches may prevent these antigens from being efficiently converted into durable adaptive immune responses.

In addition to impairing T-cell priming, adenosine-conditioned DCs may favor the expansion of regulatory or exhausted immune phenotypes. When DCs fail to provide adequate co-stimulation and inflammatory signals, tumor antigen recognition may lead to T-cell anergy, dysfunction, or incomplete activation rather than productive cytotoxic immunity. This effect can synergize with TAM- and MDSC-mediated suppression, creating a multilayered barrier that prevents effective immune surveillance. Therapeutically, reducing adenosine accumulation or blocking adenosine receptor signaling may restore DC function and improve T-cell priming. Such strategies could enhance the efficacy of cancer vaccines, STING agonists, checkpoint inhibitors, and adoptive T-cell therapies by improving antigen presentation and supporting the development of functional CTLs. Therefore, DCs represent an important but sometimes underappreciated component of adenosine-mediated immune suppression.

### 4.4 Integrated myeloid–lymphoid suppressive circuits

The effects of adenosine on TAMs, MDSCs, and DCs should not be viewed as isolated events. Rather, adenosine coordinates an integrated myeloid–lymphoid suppressive circuit. TAMs and MDSCs generate adenosine and secrete suppressive cytokines, DCs lose antigen-presenting capacity, and effector T cells become poorly primed, excluded, or exhausted. These processes reinforce one another and collectively reduce the probability of successful antitumor immune responses. This integrated model also helps explain why some tumors remain resistant to PD-1/PD-L1 blockade despite the presence of tumor antigens or partial T-cell infiltration (102, 104, 117–120). In such tumors, lymphocytes may be present but functionally constrained by adenosine-rich myeloid niches. Thus, targeting adenosine signaling may be particularly valuable in tumors with high myeloid infiltration, elevated CD39/CD73 expression, hypoxia, or poor DC activation. Combination approaches that simultaneously block classical immune checkpoints and adenosine-mediated myeloid suppression may provide a more effective strategy than targeting either pathway alone. Together, TAMs and MDSCs form a cooperative adenosine-amplifying network. TAMs contribute to adenosine-rich immune-excluded niches through CD73 expression, suppressive cytokine production, impaired antigen presentation, angiogenesis, and stromal remodeling. MDSCs further reinforce this environment by consuming extracellular ATP, producing adenosine, depleting nutrients required for T-cell function, generating ROS/NO, and inducing T-cell dysfunction or Treg expansion (86, 121–123). Through these complementary mechanisms, the adenosine pathway transforms myeloid infiltration from a potentially inflammatory response into a dominant barrier against antitumor immunity.

**Figure 3:**
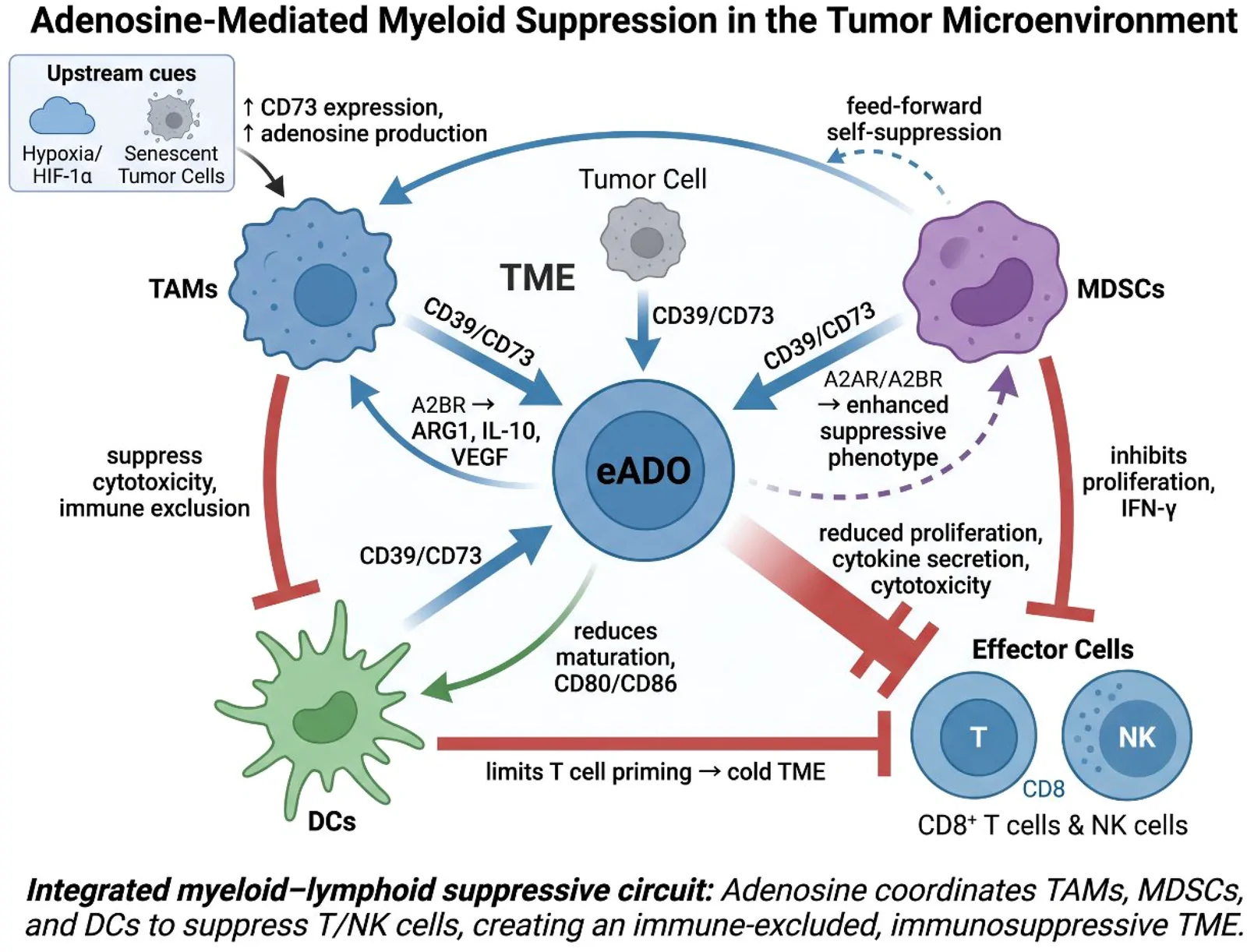
Adenosine-mediated myeloid suppression in the tumor microenvironment. CD39/CD73 on TAMs, MDSCs, DCs, and tumor cells convert ATP to extracellular adenosine (eADO). eADO inhibits CD8+ T cells and NK cells via A2AR, while A2BR signaling in TAMs and MDSCs enhances ARG1, IL-10, TGF-β, and PD-L1 expression. High adenosine also impairs DC maturation and antigen presentation, collectively creating an immune-excluded, immunosuppressive TME that promotes tumor immune evasion.

Collectively, these studies demonstrate that adenosine signaling shapes the myeloid compartment of the TME through multilayered suppression. TAMs generate and amplify adenosine to promote immune exclusion; MDSCs use the CD39/CD73–adenosine axis to directly inhibit effector lymphocytes; and DCs are functionally impaired, limiting antigen presentation and T-cell priming. By coordinating these myeloid-mediated suppressive networks, adenosine establishes a robust barrier to antitumor immunity and contributes to resistance against immune checkpoint blockade. Targeting the adenosine pathway, particularly in combination with PD-1/PD-L1 blockade, STING agonists, radiotherapy, or other immunomodulatory strategies, represents a promising approach to dismantle myeloid suppression and improve therapeutic responses. **Figure 3** illustrates how adenosine orchestrates immune suppression via lymphoid and myeloid cells. It highlights the roles of A2AR, A2BR, and DC dysfunction in shaping an immunosuppressive tumor microenvironment.

## 5 Adenosine in immune therapy resistance

Adenosine signaling not only establishes an immunosuppressive tumor microenvironment (TME), but also contributes directly to resistance against multiple forms of cancer immunotherapy. Elevated extracellular adenosine, mainly generated through CD39/CD73-mediated nucleotide metabolism, can impair effector T-cell activity, suppress NK-cell cytotoxicity, inhibit dendritic cell function, and reinforce suppressive myeloid-cell programs. As a result, adenosine-rich tumors often display features of immune exclusion, T-cell dysfunction, reduced antigen presentation, and poor responsiveness to immune-based therapies (124–127). Therapeutic resistance mediated by adenosine is particularly important because many antitumor treatments induce tissue stress, cell death, hypoxia, or inflammation, all of which may increase extracellular ATP release and subsequently promote adenosine accumulation. Although extracellular ATP can initially act as a danger signal that supports immune activation, its conversion into adenosine shifts the immune balance toward suppression. This ATP-to-adenosine transition represents a major metabolic feedback mechanism through which tumors counteract therapy-induced immune activation. Therefore, adenosine signaling should be viewed as an adaptive resistance pathway that limits the durability and magnitude of antitumor immune responses. The adaptive nature of adenosine-mediated resistance can be explained by the fact that many immune-activating therapies also generate the substrates and regulatory signals required for adenosine production. PD-1/PD-L1 blockade, STING agonists, and radiotherapy are intended to enhance T-cell activation, innate immune sensing, tumor-cell killing, antigen release, and inflammatory cytokine production (128–130). However, these same processes can increase extracellular ATP release, tissue stress, interferon-driven feedback programs, hypoxia, and myeloid-cell recruitment. Once extracellular ATP is released into a CD39/CD73-rich tumor microenvironment, it can be rapidly converted into adenosine, shifting the immune balance from activation to suppression. In this way, therapy-induced immune activation may paradoxically create a compensatory purinergic checkpoint that limits treatment efficacy. **Table 2** summarizes the key mechanisms by which adenosine mediates resistance across multiple cancer immunotherapies. It highlights how therapy-induced stress, CD39/CD73 activity, and receptor signaling converge to suppress effector immune cells and limit therapeutic efficacy.

**Table 2:** Adenosine-mediated therapy resistance mechanisms.

Therapy modality	Mechanism of resistance	Key cells/pathways	References (PMID)
PD-1/PD-L1 blockade	CD73+ exosomes generate extracellular adenosine; A2AR-mediated T cell inhibition	CD8+ T cells, NK cells, TAMs, MDSCs	(21, 23, 53)
STING agonists	Feedback upregulation of CD73 → increased adenosine; suppresses dendritic cell activation and T-cell priming	DCs, T cells	(67)
Radiotherapy/SBRT	Radiation-induced ATP release and inflammatory priming can be counterbalanced by CD39/CD73-mediated adenosine generation; CD73 blockade enhances DC infiltration, STING-dependent antitumor immunity, abscopal effects, and improves response to SBRT when combined with PD-L1 blockade	CD73, extracellular adenosine, DCs, STING, CD8^+^ T cells, PD-L1	(131–133)
Photothermal/Nanotherapy	Heat/nanoparticles increase eADO; A2AR inhibits T/NK cell activity	T cells, NK cells	(134)

### 5.1 Checkpoint inhibitors (PD-1/PD-L1)

Immune checkpoint inhibitors targeting PD-1/PD-L1 have transformed the treatment landscape of several malignancies. However, only a subset of patients achieves durable clinical benefit, and both primary and acquired resistance remain major clinical challenges. In many tumors, PD-1/PD-L1 blockade can reinvigorate exhausted T cells only when the surrounding microenvironment permits effective T-cell activation, expansion, and tumor infiltration. Adenosine-rich TMEs interfere with each of these steps, thereby limiting the efficacy of checkpoint inhibitors.

One important mechanism involves tumor-derived extracellular vesicles and exosomes. Tumor-derived exosomes enriched in CD73 can generate extracellular adenosine not only within the local tumor site but also in the peripheral circulation. This systemic adenosine production suppresses T-cell proliferation, cytokine production, and cytotoxic activity at distant sites, thereby extending tumor-mediated immune suppression beyond direct tumor–immune cell contact (135–139). Elevated serum exosomal CD73 has been associated with poor clinical response to anti-PD-1 therapy, suggesting that exosomal CD73 may function both as a mediator of resistance and as a potential predictive biomarker for immunotherapy outcomes (21). At the cellular level, adenosine signaling through A2AR and A2BR promotes T-cell dysfunction and exhaustion. Engagement of A2AR on cytotoxic T lymphocytes suppresses TCR signaling, reduces IFN-γ and TNF-α production, impairs perforin- and granzyme-dependent cytotoxicity, and limits proliferative capacity. Persistent exposure to adenosine may also cooperate with PD-1 signaling to deepen the exhausted phenotype of T cells. In this setting, blockade of PD-1 alone may be insufficient because the adenosine pathway continues to impose a metabolic brake on effector function.

A2BR signaling further contributes to checkpoint inhibitor resistance by regulating the myeloid compartment. In adenosine-rich tumors, A2BR activation in TAMs and MDSCs promotes suppressive cytokine production, weakens antigen presentation, and enhances T-cell exclusion. These myeloid-mediated effects create a microenvironment in which checkpoint blockade cannot fully restore antitumor immunity. Experimental evidence indicates that inhibition of A2AR or A2BR can restore CTL function and improve the efficacy of PD-1 blockade, supporting the concept that adenosine receptor signaling represents a parallel and complementary resistance mechanism to classical immune checkpoints (23, 53). Thus, the CD73–adenosine–A2AR/A2BR axis provides a strong rationale for combination strategies. In tumors with high CD73 expression, elevated extracellular adenosine, abundant suppressive myeloid cells, or poor T-cell infiltration, combining PD-1/PD-L1 inhibitors with CD39/CD73 blockade or adenosine receptor antagonists may be more effective than checkpoint inhibition alone. This approach aims to remove both the classical inhibitory receptor signal and the metabolic suppressive barrier. In the setting of PD-1/PD-L1 blockade, adenosine-mediated suppression may emerge as an adaptive escape mechanism because reinvigorated T cells increase inflammatory cytokine production and tumor-cell killing, thereby increasing extracellular nucleotide release and local tissue stress (140, 141). At the same time, tumors with high CD73 expression, CD39-positive regulatory or myeloid cells, or CD73-enriched exosomes can convert this inflammatory ATP-rich environment into an adenosine-dominant niche. Persistent A2AR signaling then restrains the cytotoxic program of reactivated T cells, while A2BR signaling reinforces TAM/MDSC-mediated suppression. Therefore, PD-1/PD-L1 blockade may fail not because T cells cannot be initially reinvigorated, but because the adenosine axis imposes a parallel metabolic brake that limits sustained effector function.

### 5.2 STING agonists

The STING pathway is a central innate immune sensing mechanism that detects cytosolic DNA and triggers type I interferon production. Activation of STING can promote dendritic cell maturation, enhance cross-presentation of tumor antigens, recruit effector T cells, and convert immunologically “cold” tumors into more inflamed lesions (142–146). For this reason, STING agonists have attracted considerable interest as immunotherapeutic agents, particularly in combination with immune checkpoint blockade.

However, recent experimental findings suggest that STING activation can also induce compensatory immunosuppressive feedback. One important feedback mechanism involves the upregulation of the CD73–adenosine axis. Although STING agonists initially activate innate immune signaling, subsequent induction of CD73 can increase extracellular adenosine accumulation in the TME. This adenosine-rich state suppresses dendritic cell activation, weakens T-cell priming, and limits the full antitumor potential of STING pathway activation (67). This feedback loop has important therapeutic implications. STING agonist treatment may generate both immune-stimulatory and immune-suppressive signals at the same time. On the one hand, STING activation promotes type I interferon signaling and antitumor immunity; on the other hand, CD73-mediated adenosine production can restrain this immune activation and prevent durable tumor control. This may help explain why STING agonists do not always produce robust antitumor responses as monotherapies, especially in tumors with pre-existing hypoxia, high CD73 expression, or myeloid-dominant immunosuppression. Mechanistically, this represents a negative-feedback loop between innate immune activation and purinergic suppression. STING activation promotes type I interferon production, dendritic-cell activation, antigen cross-presentation, and T-cell recruitment. However, the resulting inflammatory and interferon-rich environment may also induce CD73 expression or enhance the functional relevance of pre-existing CD73-positive tumor, stromal, or myeloid compartments (26, 39). As a result, the same STING-dependent inflammation that initiates antitumor immunity can also promote adenosine accumulation, which suppresses dendritic-cell function and limits durable T-cell priming. Combining STING agonists with adenosine-axis blockade may therefore represent a rational strategy. CD73 inhibition or A2AR/A2BR antagonism could preserve the beneficial interferon-driven effects of STING activation while preventing adenosine-mediated suppression of dendritic cells and T cells. Such combinations may be particularly useful in tumors that require innate immune activation but rapidly develop compensatory metabolic immune resistance.

### 5.3 Radiotherapy and other cytotoxic local therapies

Radiotherapy and other cytotoxic local therapies can initiate antitumor immunity by inducing tumor-cell death, antigen release, inflammatory danger signals, and dendritic-cell-dependent T-cell priming (147–149). However, these immune-stimulatory effects may be counterbalanced by compensatory purinergic metabolism. Radiation-induced tissue injury and tumor-cell stress increase the availability of extracellular ATP, which can initially act as a danger-associated molecular pattern but is subsequently hydrolyzed by CD39 and CD73 into immunosuppressive adenosine. This ATP-to-adenosine conversion is particularly relevant after radiotherapy because irradiated tumors contain dying tumor cells, damaged stromal cells, inflammatory infiltrates, and hypoxic tissue regions, all of which can favor purinergic remodeling. Thus, radiotherapy can simultaneously provide immunogenic priming and create the metabolic substrate for CD39/CD73-dependent adenosine generation. Therefore, adenosine-axis blockade is mechanistically complementary to radiotherapy: radiotherapy provides antigenic and inflammatory priming, whereas CD73 or adenosine receptor inhibition prevents the conversion of this inflammatory response into an adenosine-dominated suppressive niche (150). Direct experimental evidence supports this combination strategy. Wennerberg et al. demonstrated that CD73 blockade enhanced dendritic cell infiltration in irradiated tumors and promoted tumor rejection, indicating that the CD73–adenosine axis can limit radiotherapy-induced immune priming (131). More recently, An et al. showed that CD73 blockade potentiated radiotherapy-induced antitumor immunity and abscopal effects through activation of the STING pathway (133). In murine pancreatic ductal adenocarcinoma models, Ye et al. further demonstrated that dual blockade of CD73 and PD-L1 amplified the antitumor efficacy of stereotactic body radiotherapy, remodeled the immunosuppressive tumor microenvironment toward a more immunostimulatory state, generated long-term antitumor memory, and improved systemic control of metastatic disease (132). These studies collectively suggest that CD73/adenosine blockade is not merely an additive partner to radiotherapy, but may function as a mechanism-based strategy to preserve radiation-induced innate and adaptive immune activation while preventing adenosine-mediated adaptive immune resistance.

A similar principle may apply to other cytotoxic local therapies, including photothermal and nanomaterial-based treatments, where tumor destruction can release antigens and inflammatory mediators but may also generate metabolic stress that favors adenosine accumulation. In this context, nanocarriers capable of delivering CD73 inhibitors, A2AR/A2BR antagonists, or immune adjuvants may help synchronize tumor-cell killing with metabolic checkpoint blockade (134, 151). Nevertheless, compared with radiotherapy, the evidence for photothermal or nanomaterial-based adenosine modulation remains more preclinical and platform-dependent; therefore, these approaches should be discussed as emerging strategies rather than as established radiotherapy-like combination paradigms.

### 5.4 Adenosine as an adaptive resistance program across modalities

Across checkpoint blockade, STING agonism, radiotherapy, and other cytotoxic immune-activating therapies, a common adaptive pattern emerges: the stronger the therapy-induced inflammatory or cytotoxic response, the greater the potential for extracellular nucleotide release, CD39/CD73-dependent adenosine production, and A2AR/A2BR-mediated immune suppression. This explains why adenosine signaling can function as an inducible resistance program rather than merely a pre-existing suppressive pathway. In this model, therapy-induced tumor injury and immune activation generate extracellular ATP and inflammatory signals, whereas the purinergic machinery of the TME converts these signals into an adenosine-mediated negative feedback loop (152–155). This suggests that the adenosine axis functions as a general adaptive resistance program rather than a resistance mechanism limited to a single therapeutic class. The pathway becomes particularly relevant under conditions of hypoxia, cell stress, tissue injury, and extracellular nucleotide release, all of which are common consequences of anticancer treatment. This adaptive resistance model has practical implications for treatment design. First, baseline CD39/CD73 expression, adenosine receptor expression, exosomal CD73 levels, and myeloid-cell infiltration may help identify patients who are less likely to respond to monotherapy (36, 38). Second, dynamic changes in adenosine-related biomarkers during treatment may reveal early emergence of resistance. Third, timing may be critical: adenosine-axis blockade may need to be administered concurrently with, or shortly after, immune-activating therapies to prevent the establishment of suppressive feedback loops. Overall, adenosine-driven resistance reflects the metabolic plasticity of the TME. Tumors do not simply evade immunity through fixed checkpoint expression; they dynamically convert inflammatory and therapeutic stress signals into suppressive metabolites. This highlights the need to integrate metabolic checkpoint blockade into next-generation immunotherapy combinations.

**Figure 4:**
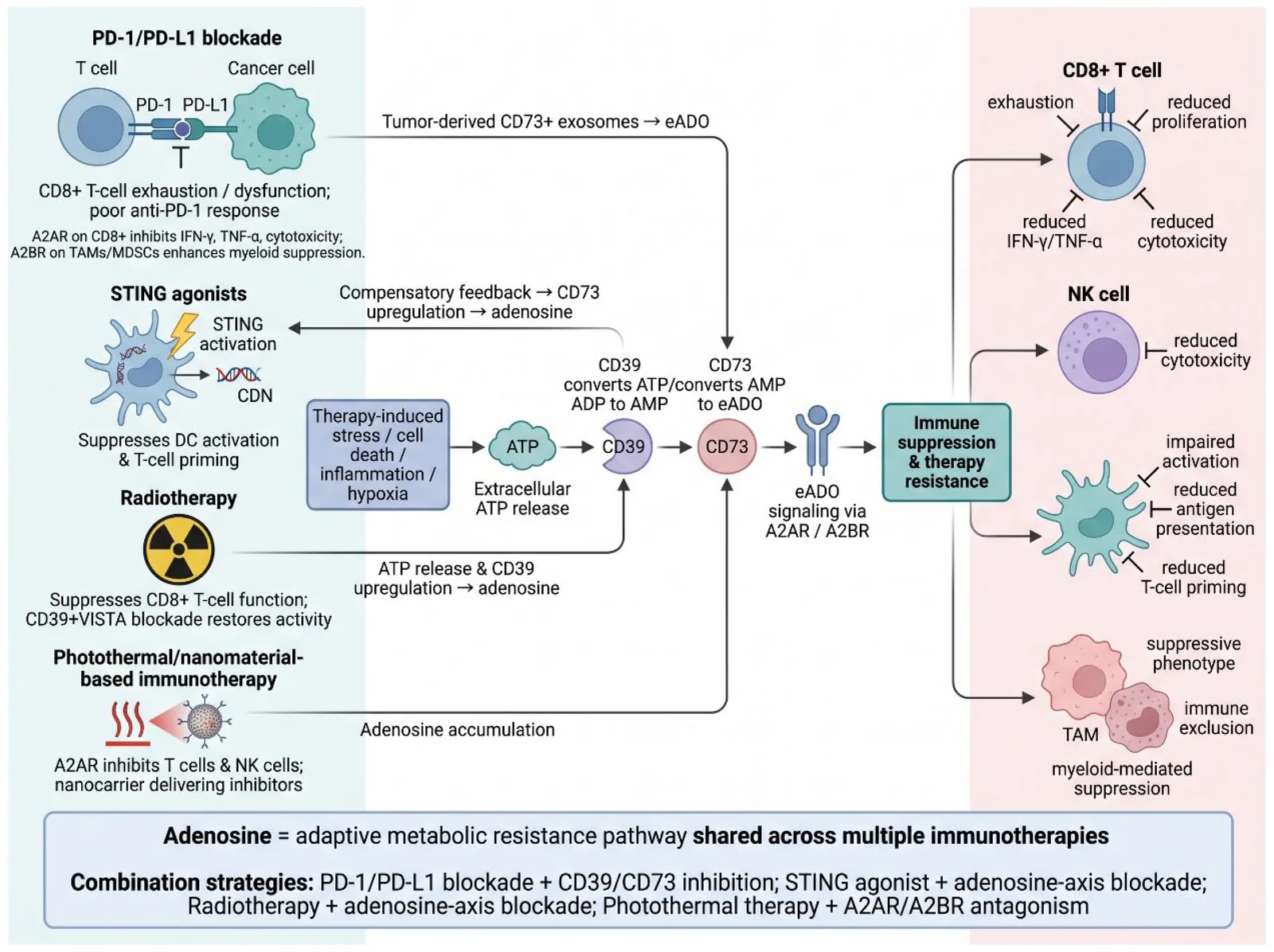
Adenosine-mediated adaptive resistance in cancer immunotherapy. Therapy-induced tumor stress releases extracellular ATP, which is converted to adenosine by CD39/CD73. Adenosine suppresses CD8+ T cells and NK cells through A2AR, while A2BR signaling in TAMs and MDSCs promotes immunosuppressive cytokines and PD-L1. Adenosine also inhibits dendritic cell activation and T-cell priming, collectively contributing to primary and acquired resistance to PD-1/PD-L1 blockade, STING agonists, radiotherapy, and photothermal or nanomaterial-based therapies.

Collectively, these studies illustrate that the adenosine axis is a central barrier to effective immunotherapy across multiple treatment modalities. CD73-mediated adenosine production contributes to primary and acquired resistance to PD-1/PD-L1 blockade, STING agonists, radiotherapy, and photothermal or nanomaterial-based therapies. Mechanistically, adenosine suppresses effector lymphocytes, promotes T-cell exhaustion, impairs dendritic cell activation, and reinforces myeloid-driven immunosuppression. These insights provide a strong rationale for combination therapeutic strategies, such as checkpoint blockade plus CD39/CD73 inhibition or A2AR/A2BR antagonism, to overcome resistance and improve clinical outcomes. Future work should define which tumors are most dependent on adenosine-mediated resistance and determine the optimal timing, biomarkers, and combination partners for adenosine-targeted therapy. **Figure 4** illustrates how adenosine integrates multiple mechanisms to create an immunosuppressive tumor microenvironment, highlighting its role in lymphoid and myeloid suppression and therapy resistance.

## 6 Therapeutic targeting of the adenosine axis

Given the central role of the CD39/CD73–adenosine–A2AR/A2BR axis in tumor immune evasion and therapy resistance, therapeutic targeting of this pathway has become an important strategy in cancer immunotherapy. The biological rationale is straightforward: reducing extracellular adenosine production or blocking its downstream receptors can release metabolic constraints on antitumor immunity, restore effector lymphocyte function, and weaken myeloid-mediated immunosuppression. Unlike classical immune checkpoint blockade, which primarily targets inhibitory receptor–ligand interactions, adenosine-axis targeting aims to remodel the metabolic and immunological landscape of the tumor microenvironment (TME). Multiple therapeutic approaches are currently being explored, including inhibition of ectonucleotidases, blockade of adenosine receptors, antibody-based targeting of CD39 or CD73, engineered immune-cell strategies, and nanoparticle-based drug delivery (156–158). These approaches are not mutually exclusive. In fact, because adenosine signaling affects tumor cells, T cells, NK cells, TAMs, MDSCs, DCs, and stromal elements, combination strategies may be required to achieve durable antitumor immunity. The therapeutic goal is not simply to reduce adenosine concentration, but to convert an adenosine-rich, myeloid-dominant, immune-excluded TME into an immune-permissive environment capable of supporting T-cell priming, infiltration, and cytotoxic function.

### 6.1 CD39/CD73 inhibition

Direct inhibition of CD39 and CD73 represents a source-targeting approach because these ectonucleotidases are responsible for the sequential conversion of extracellular ATP and ADP into AMP and adenosine. In tumors, ATP released by stressed, dying, or therapy-damaged cells can initially act as an immunostimulatory danger signal. However, when CD39 and CD73 are highly expressed, extracellular ATP is rapidly degraded into adenosine, shifting the immune balance from activation to suppression. Therefore, blocking CD39/CD73 may preserve ATP-mediated inflammatory signaling while reducing adenosine-mediated immune inhibition.

CD73 is one of the most extensively investigated targets in this pathway. CD73 inhibitors and monoclonal antibodies can reduce extracellular adenosine generation and restore immune effector function (159–162). Antibody-based CD73 blockade may exert several complementary effects: enzymatic inhibition, reduction of adenosine accumulation, enhancement of NK-cell cytotoxicity, modulation of B-cell responses, and improvement of T-cell-mediated tumor control. For example, anti-CD73 antibodies such as IBI325 have shown the ability to inhibit CD73 activity and restore antitumor immune responses in preclinical or translational settings (69, 70). These findings support CD73 as both a metabolic checkpoint and a therapeutic entry point for reversing adenosine-driven suppression.

Importantly, CD73 is expressed not only on tumor cells but also on stromal cells, endothelial cells, regulatory T cells, TAMs, MDSCs, and extracellular vesicles. This broad expression pattern suggests that CD73 blockade may influence multiple compartments of the TME. In addition to reducing tumor-cell-derived adenosine, CD73 inhibition may weaken suppressive immune-cell networks and improve immune-cell trafficking into tumor tissues. This is particularly relevant in tumors with high CD73 expression, strong hypoxic features, abundant myeloid infiltration, or poor response to PD-1/PD-L1 blockade. CD39 inhibition offers a complementary strategy. As the upstream ectonucleotidase that hydrolyzes ATP and ADP into AMP, CD39 controls the balance between proinflammatory extracellular ATP signaling and immunosuppressive adenosine production. Targeting CD39 may therefore preserve ATP-dependent immune activation while limiting downstream adenosine accumulation. Anti-CD39 antibodies have been shown to reduce suppressive cellular populations within the TME, including MDSCs and regulatory T cells, thereby facilitating T-cell-mediated tumor eradication (68). Compared with CD73 inhibition alone, CD39 blockade may have broader effects on purinergic signaling by preventing the initial degradation of extracellular ATP.

However, the therapeutic consequences of CD39/CD73 inhibition may depend on tumor context. In some tumors, CD73 on malignant cells may be the dominant source of adenosine; in others, myeloid cells, Tregs, stromal cells, or exosomes may contribute more substantially. Therefore, patient selection based on CD39/CD73 expression patterns, spatial localization, and cellular source will be essential. Future therapeutic design should consider whether the goal is to block tumor-intrinsic CD73, suppress myeloid-derived adenosine, preserve ATP-mediated immune activation, or achieve broad remodeling of the purinergic TME. Thus, the main conceptual strength of CD39/CD73 inhibition is upstream source control of adenosine production, but its clinical success will depend on whether the targeted enzyme is the dominant and spatially relevant source of immunosuppressive adenosine in a given tumor.

### 6.2 A2AR/A2BR antagonists

Another major strategy is to block adenosine receptors on immune and stromal cells. Among the four adenosine receptors, A2AR and A2BR are most closely linked to tumor immune suppression. A2AR is highly relevant in effector T cells and NK cells, whereas A2BR is frequently associated with myeloid cells, stromal components, and hypoxic tumor environments. Blocking these receptors can prevent adenosine from transmitting suppressive signals even when extracellular adenosine remains abundant.

A2AR antagonism primarily aims to restore lymphocyte function. In cytotoxic T lymphocytes, A2AR signaling suppresses TCR activation, reduces cytokine production, limits proliferation, and impairs perforin- and granzyme-mediated killing. In NK cells, A2AR activation similarly weakens cytotoxicity and antitumor surveillance. Pharmacological blockade of A2AR can restore CTL and NK-cell function by preventing adenosine-mediated inhibition of proliferation, IFN-γ production, and cytotoxic effector programs (52, 53). These effects are particularly important in tumors where T cells are present but functionally restrained by metabolic suppression. A2BR antagonism may be especially valuable for targeting myeloid-driven immune resistance. A2BR signaling in TAMs and MDSCs can enhance suppressive cytokine production, promote ARG1 and IL-10 expression, impair antigen presentation, and support angiogenesis or stromal remodeling. Therefore, A2BR blockade may reduce myeloid-mediated immunosuppression and improve T-cell access to tumor tissue. This receptor is also closely linked to hypoxic TMEs, where adenosine levels are often high and A2BR expression may be functionally important.

Emerging dual A2A/A2B receptor antagonists, such as M1069, aim to inhibit both lymphoid and myeloid arms of adenosine signaling. This approach is conceptually attractive because A2AR and A2BR have complementary roles in the TME. A2AR blockade can reinvigorate effector T cells and NK cells, whereas A2BR blockade may attenuate suppressive myeloid-cell programming. Preclinical studies suggest that dual receptor antagonism can enhance cytotoxic T-cell activity, reduce myeloid-mediated suppression, and improve antitumor responses when combined with other therapies (163). The choice between enzyme inhibition and receptor antagonism remains an important therapeutic question. CD39/CD73 inhibitors reduce adenosine generation at the source, whereas A2AR/A2BR antagonists block downstream signaling. Enzyme inhibition may be more effective when adenosine production is localized and targetable, while receptor blockade may be useful when adenosine is generated from multiple sources or when complete reduction of extracellular adenosine is difficult. In some settings, combining enzyme inhibition with receptor antagonism may provide a deeper blockade of the pathway, although toxicity, redundancy, and systemic immune effects need careful evaluation. Thus, the main conceptual strength of receptor antagonism is downstream signal interception, particularly when adenosine is produced by multiple or poorly defined sources; however, receptor blockade may be incomplete if several adenosine receptors or non-receptor adenosine-dependent mechanisms remain active.

### 6.3 Enzyme inhibition versus receptor antagonism: theoretical advantages and limitations

Direct targeting of CD39 or CD73 and blockade of A2AR/A2BR represent two mechanistically distinct strategies for inhibiting the adenosine pathway. CD39/CD73 inhibition acts upstream by reducing extracellular adenosine generation at its enzymatic source. The theoretical advantage of this approach is that it may broadly reduce adenosine availability before adenosine can activate multiple receptors on different immune, stromal, and tumor-cell compartments. CD39 inhibition may also preserve extracellular ATP, which can function as an immunostimulatory danger signal and support dendritic-cell activation, inflammasome activity, and T-cell priming. CD73 blockade may be particularly useful in tumors with high CD73 expression on malignant cells, myeloid cells, stromal cells, endothelial cells, or extracellular vesicles, where local adenosine generation is a dominant suppressive mechanism (164–166).

However, enzyme inhibition also has limitations. CD39 and CD73 are expressed across multiple cell types, and total expression does not necessarily reflect functional enzymatic activity or spatially relevant adenosine production. In some tumors, adenosine may arise from multiple cellular sources or alternative metabolic routes, making single-enzyme inhibition incomplete. CD39 blockade may increase extracellular ATP accumulation, which can be immunostimulatory but may also contribute to inflammation or tissue injury depending on context. CD73 blockade may be less effective if adenosine is generated by CD73-independent pathways or if downstream receptor signaling remains highly sensitive to residual adenosine (85, 136). Therefore, CD39/CD73 inhibitors may require biomarker-based selection according to enzymatic activity, cellular source, spatial localization, and extracellular nucleotide availability.

By contrast, A2AR/A2BR antagonists act downstream by preventing extracellular adenosine from transmitting suppressive signals through its receptors. The theoretical advantage of receptor blockade is that it can inhibit adenosine signaling even when adenosine is produced by multiple tumor, stromal, myeloid, or exosomal sources. A2AR antagonism may be particularly useful for restoring metabolically inhibited CD8+ T-cell and NK-cell function, whereas A2BR blockade may be more relevant in myeloid-rich, hypoxic, or immune-excluded tumors where TAMs and MDSCs dominate the suppressive landscape (20, 58). Receptor antagonists may also allow more selective targeting of lymphoid versus myeloid mechanisms depending on receptor expression patterns.

Nevertheless, receptor blockade also has disadvantages. It does not reduce extracellular adenosine itself and therefore may not restore ATP-mediated immune activation or prevent adenosine from acting through unblocked receptors. Blocking A2AR alone may be insufficient in tumors where A2BR-driven myeloid suppression is dominant, whereas A2BR blockade alone may not fully restore T/NK-cell cytotoxicity if A2AR signaling remains active. In addition, A2AR and A2BR are expressed in normal tissues and participate in vascular, inflammatory, and tissue-protective physiology, raising potential concerns about systemic toxicity and therapeutic window. Overall, enzyme inhibition may be preferable when a tumor contains spatially localized, CD39/CD73-dependent adenosine production, whereas receptor antagonism may be advantageous when adenosine is generated by heterogeneous sources but suppresses defined A2AR- or A2BR-expressing immune populations.

### 6.4 Combination immunotherapy strategies

Because adenosine-mediated suppression intersects with multiple immune resistance pathways, combination therapy is likely to be the most effective clinical strategy. Adenosine-axis inhibitors may be combined with PD-1/PD-L1 blockade, CTLA-4 blockade, STING agonists, radiotherapy, chemotherapy, photothermal therapy, or adoptive cell therapy. The central principle is to pair immune activation with metabolic checkpoint removal. In this framework, PD-1/PD-L1 blockade and adenosine-axis inhibition act on distinct but convergent barriers to antitumor immunity (167–169). Checkpoint blockade restores antigen-driven T-cell activation, whereas adenosine-axis inhibition prevents extracellular adenosine from suppressing reactivated lymphocytes and from sustaining TAM/MDSC-mediated immune exclusion. This provides the mechanistic basis for combining these approaches rather than relying on either strategy alone. While checkpoint inhibitors can reinvigorate exhausted T cells, adenosine blockade can remove a parallel metabolic brake that limits cytotoxic function and immune-cell infiltration.

Combining CD39/CD73 inhibition with PD-1/PD-L1 blockade is one of the most rational approaches. In adenosine-rich tumors, PD-1 blockade alone may fail because T cells remain metabolically suppressed through A2AR signaling and because myeloid populations continue to maintain an immune-excluded niche. Inhibition of CD39/CD73 can reduce adenosine production, enhance effector T-cell function, and improve responsiveness to checkpoint blockade. Preclinical findings support the ability of CD39/CD73 targeting to reinvigorate exhausted T cells and enhance antitumor immunity when combined with checkpoint inhibitors (105). The adenosine axis is also relevant to STING agonist therapy. STING activation can induce type I interferon responses, promote dendritic cell activation, and improve T-cell priming. However, STING agonists may also induce compensatory CD73 upregulation and adenosine accumulation, thereby limiting the durability of immune activation. Combining STING agonists with CD73 inhibition or adenosine receptor antagonists may preserve the immune-stimulatory benefits of STING activation while preventing adenosine-mediated negative feedback (67). This strategy may be especially useful in tumors that are poorly inflamed but capable of innate immune activation.

Radiotherapy provides another important combination partner. Radiation can induce immunogenic cell death, increase antigen release, and promote inflammatory signaling, but it can also enhance extracellular nucleotide metabolism and adenosine accumulation. In this context, CD39/CD73 or adenosine receptor blockade may prevent radiation-induced immune suppression and help transform local tumor damage into systemic antitumor immunity. The combination of CD39 blockade with other immune modulators, such as VISTA inhibition, has been shown to restore CD8+ T-cell activity and overcome therapy-induced suppressive feedback (105). Adoptive cell therapies may also benefit from adenosine-axis targeting. CAR-T, TIL, and CAR-NK therapies require immune effector cells to survive, infiltrate, and function within hostile tumor tissues. However, high extracellular adenosine can suppress cytotoxicity, cytokine secretion, and persistence of transferred cells. Strategies that target CD73 on tumor cells or block A2AR signaling in engineered immune cells may improve adoptive cell therapy performance. CD73-directed CAR-NK strategies are particularly attractive because they may simultaneously kill CD73-positive tumor cells and reduce local adenosine-mediated suppression (44).

### 6.5 Nanoparticle-based and local delivery approaches

Nanoparticle-based delivery systems provide an additional layer of therapeutic innovation. Systemic blockade of purinergic signaling may raise concerns about off-target immune activation, cardiovascular effects, or disruption of physiological adenosine functions in normal tissues. Nanocarriers can improve tumor selectivity, increase local drug accumulation, provide controlled release, and enable co-delivery of multiple therapeutic agents. This is particularly valuable for adenosine-targeted therapy because adenosine-mediated suppression is often spatially restricted to hypoxic and metabolically stressed tumor regions.

Nanoparticle platforms can be engineered to deliver CD39/CD73 inhibitors, A2AR/A2BR antagonists, immune adjuvants, photosensitizers, or oxygen-generating components. For example, ROS-responsive or tumor-microenvironment-responsive nanocarriers may release drugs selectively within tumors, thereby enhancing immune activation while limiting systemic toxicity. Nanomaterial-based strategies can also combine photothermal therapy with adenosine-axis blockade, allowing local tumor destruction and simultaneous relief of metabolic immune suppression (134, 170). Such systems are especially relevant in metabolically hostile TMEs characterized by hypoxia, acidosis, lactate accumulation, and high adenosine levels. By improving intratumoral delivery and coordinating immune activation with metabolic checkpoint blockade, nanoparticle-based therapies may overcome several limitations of conventional systemic therapy. However, translation will require careful optimization of nanoparticle size, biodistribution, drug-release kinetics, immunogenicity, safety, and manufacturing reproducibility.

### 6.6 Translational obstacles and biomarker-guided patient selection

Despite strong biological rationale, several translational obstacles may limit the clinical application of adenosine-pathway inhibitors. First, adenosine signaling is highly context-dependent. The dominant source of extracellular adenosine may differ across tumor types and may arise from malignant cells, stromal cells, endothelial cells, Tregs, TAMs, MDSCs, dendritic cells, or extracellular vesicles. Therefore, total CD73 expression alone may not accurately identify tumors that are functionally dependent on the adenosine pathway. Second, adenosine has important physiological roles in tissue protection, vascular regulation, and inflammation resolution; systemic blockade of CD73 or A2AR may therefore raise concerns about off-target toxicity, altered inflammatory responses, or organ-specific adverse effects (25, 171). Third, redundancy within purinergic and immunosuppressive networks may reduce the efficacy of single-agent adenosine blockade. Even when CD73 or A2AR is inhibited, compensatory pathways such as PD-1/PD-L1, CTLA-4, VISTA, TGF-β, IDO1, lactate metabolism, hypoxia-driven programs, and suppressive myeloid-cell recruitment may continue to maintain immune resistance. Fourth, the optimal timing, sequence, and combination partners remain unresolved, particularly in settings where chemotherapy or radiotherapy may first increase extracellular ATP release and then promote CD39/CD73-dependent adenosine accumulation.

Biomarker-guided patient selection will therefore be essential. Candidate predictive biomarkers include high tumor-cell or stromal CD73 expression, CD39 expression on Tregs or suppressive myeloid cells, A2AR expression on exhausted CD8+ T cells and NK cells, A2BR expression on TAMs and MDSCs, elevated circulating or exosomal CD73, increased intratumoral adenosine levels, high AMP/adenosine ratios, hypoxia or lactate-related metabolic signatures, and abundant TAM/MDSC infiltration (172, 173). Spatial biomarkers may be particularly informative, because the biological effect of adenosine depends not only on its abundance but also on where it is produced. For example, tumors with CD73-positive TAMs or stromal cells located near excluded or exhausted T cells may be more likely to benefit from adenosine-axis blockade than tumors with low, diffuse, or spatially disconnected CD73 expression.

In addition to baseline predictive biomarkers, pharmacodynamic biomarkers are needed to confirm target engagement and biological activity during treatment. These may include reduced CD73 enzymatic activity, decreased intratumoral or circulating adenosine concentration, altered ATP/AMP/adenosine balance, reduced A2AR/A2BR-associated suppressive transcriptional signatures, increased dendritic-cell activation, enhanced CD8+ T-cell infiltration, increased IFN-γ and granzyme B expression, and decreased TAM/MDSC suppressive signatures (174, 175). Integrating baseline predictive markers with on-treatment pharmacodynamic readouts may help identify patients most likely to benefit from anti-CD73 antibodies, A2AR/A2BR antagonists, or combination strategies with immune checkpoint blockade, chemoradiotherapy, radiotherapy, or adoptive cell therapy. **Table 3** outlines current and potential therapeutic strategies targeting the adenosine axis in combination with immune checkpoint blockade, STING agonists, radiotherapy, or nanomaterial-based therapies. It emphasizes mechanistic rationale and expected benefits for overcoming adaptive immune resistance.

**Table 3:** Translational and combination therapy strategies.

Strategy	Combination/approach	Mechanistic rationale	Potential benefit	References (PMID)
PD-1/PD-L1 + CD39/CD73 inhibition	Checkpoint + metabolic blockade	Reduces adenosine-mediated T cell suppression	Overcomes primary/acquired resistance	(105)
STING agonist + CD73/A2AR/A2BR blockade	Innate activation + adenosine suppression	Prevents feedback suppression; enhances DC and T-cell priming	Improved tumor control	(67)
Radiotherapy/SBRT + CD73 blockade	RT or SBRT combined with anti-CD73, with or without PD-L1 blockade	RT induces antigen release and inflammatory priming, whereas CD73 blockade prevents ATP-to-adenosine conversion and preserves DC/STING/CD8^+^ T-cell activation	Enhances tumor rejection, promotes abscopal effects, remodels the TME toward an immunostimulatory phenotype, and may improve systemic disease control	(131, 132, 133)
Photothermal/Nanoparticles + A2AR/A2BR antagonists	Local stress + receptor blockade	Blocks A2AR-mediated T/NK suppression	Enhances immune amplification	(134)
Post-cCRT consolidation immunotherapy + CD73 blockade	Durvalumab + oleclumab after concurrent chemoradiotherapy in unresectable stage III NSCLC	cCRT induces tumor stress, antigen release, and extracellular nucleotide release; CD73 blockade may prevent adenosine-mediated suppression and enhance PD-L1 blockade	May prolong PFS and improve consolidation immunotherapy efficacy; being evaluated in phase III PACIFIC-9	(176, 177)

## 7 Translational and clinical implications

For clinical translation, adenosine-related biomarkers should not be viewed simply as static markers of CD73 expression, but rather as functional indicators of purinergic metabolism, adenosine generation, receptor-mediated immune suppression, and pharmacodynamic pathway inhibition. Biomarker development should therefore distinguish predictive biomarkers that identify tumors likely to depend on the CD39/CD73–adenosine axis from pharmacodynamic biomarkers that confirm effective target engagement after treatment (24, 178). This distinction is particularly important for combination strategies involving checkpoint blockade and chemoradiotherapy, where cytotoxic tumor injury may increase extracellular ATP release and subsequently drive CD39/CD73-dependent adenosine accumulation. The growing body of evidence on adenosine signaling in the tumor microenvironment (TME) highlights its critical translational and clinical relevance. Both the enzymatic machinery responsible for adenosine generation—CD39 and CD73—and downstream receptor-mediated pathways, including A2AR and A2BR, are actionable targets for cancer therapy. Adenosine not only suppresses effector lymphocytes but also orchestrates myeloid-mediated immune evasion, providing a mechanistic basis for its consideration as a metabolic checkpoint. Several studies suggest that adenosine-related biomarkers can inform patient stratification, guide therapeutic decisions, and potentially predict responses to immune-based therapies.

### 7.1 CD73 and extracellular adenosine as predictive biomarkers

High tumor or stromal CD73 expression, elevated CD39 expression on immunosuppressive immune subsets, increased extracellular adenosine levels, and circulating exosomal CD73 may serve as candidate predictive biomarkers for identifying adenosine-dependent immune suppression (35, 179). These markers are biologically relevant because they reflect different layers of the pathway: ATP-to-AMP conversion by CD39, AMP-to-adenosine conversion by CD73, systemic adenosine-generating capacity of extracellular vesicles, and downstream suppression of effector immune cells through A2AR/A2BR signaling. However, CD73 immunohistochemistry alone is unlikely to be sufficient for patient selection, because CD73 can be expressed by tumor cells, endothelial cells, fibroblasts, Tregs, TAMs, MDSCs, and extracellular vesicles. Therefore, biomarker interpretation should consider not only the level of CD73 expression, but also its cellular source, spatial localization, enzymatic activity, and relationship to exhausted T cells or suppressive myeloid niches (64, 180). In addition to predictive biomarkers, pharmacodynamic biomarkers are needed to determine whether adenosine-axis blockade is biologically active in patients. Potential pharmacodynamic readouts include reduced CD73 enzymatic activity, decreased intratumoral or circulating adenosine levels, altered AMP/adenosine ratios, reduced A2AR/A2BR-associated suppressive transcriptional signatures, increased dendritic-cell activation, enhanced CD8+ T-cell infiltration, increased IFN-γ/granzyme B expression, and decreased suppressive TAM or MDSC signatures (181–183). In trials combining adenosine modulation with chemoradiotherapy or immune checkpoint blockade, longitudinal sampling before and after treatment may be particularly informative for distinguishing baseline adenosine dependence from therapy-induced compensatory adenosine accumulation. For clinical implementation, these biomarkers should be interpreted according to adenosine biology rather than as isolated expression markers. A useful biomarker strategy should determine whether the tumor has sufficient extracellular nucleotide substrate, active CD39/CD73 enzymatic conversion, receptor-expressing immune targets, and spatially organized suppressive niches. Thus, the most informative patient-selection model may combine tumor or stromal CD73 expression, CD39-positive suppressive immune populations, A2AR/A2BR expression on relevant immune subsets, exosomal CD73, hypoxia/lactate signatures, and spatial proximity between adenosine-producing cells and dysfunctional T/NK cells. Such a multi-layered approach may better identify adenosine-dependent tumors than CD73 immunohistochemistry alone.

### 7.2 Combining adenosine-targeted therapy with immune checkpoint blockade

Preclinical and early clinical studies demonstrate that adenosine-mediated immunosuppression can significantly undermine the efficacy of conventional immune checkpoint inhibitors. The persistent presence of adenosine promotes T cell exhaustion, reduces cytokine production, and limits cytotoxicity, thereby attenuating responses to PD-1/PD-L1 blockade (23, 53). Targeting adenosine signaling at multiple levels—either through CD39/CD73 inhibition to reduce adenosine production, or through A2AR/A2BR blockade to prevent adenosine receptor signaling—can restore T cell effector functions, reinvigorate exhausted T cells, and enhance tumor-infiltrating lymphocyte activity. Combining these approaches with immune checkpoint blockade offers a rational strategy to overcome both intrinsic resistance (pre-existing immune suppression) and acquired resistance (therapy-induced adaptation), expanding the proportion of patients who can benefit from immunotherapy. The clinical development of oleclumab, an anti-CD73 monoclonal antibody, provides an important example of how adenosine biology can be translated into rational combination therapy. In the COAST randomized phase II trial, patients with unresectable stage III NSCLC who had not progressed after definitive platinum-based concurrent chemoradiotherapy were treated with consolidation durvalumab alone or durvalumab combined with oleclumab or monalizumab. The updated analysis showed that durvalumab plus oleclumab was associated with numerically higher objective response rate and prolonged progression-free survival compared with durvalumab alone, with no new or notable safety signals, supporting further evaluation of this approach in larger trials (176). Importantly, this clinical setting is biologically relevant to adenosine modulation because chemoradiotherapy can generate tumor-cell stress, antigen release, inflammatory priming, and extracellular nucleotide release, whereas CD73 blockade may prevent the downstream accumulation of immunosuppressive adenosine. This rationale is now being tested in PACIFIC-9, an international, randomized, double-blind phase III trial comparing durvalumab plus oleclumab or durvalumab plus monalizumab with durvalumab plus placebo in patients with unresectable stage III NSCLC who have not progressed after definitive platinum-based concurrent chemoradiotherapy (177). The PACIFIC-9 design highlights a clinically actionable strategy in which adenosine-axis inhibition is not used as monotherapy, but as a consolidation partner for PD-L1 blockade after chemoradiotherapy. From a biomarker perspective, such trials provide an opportunity to evaluate whether baseline CD73 expression, soluble or exosomal CD73, hypoxia-related signatures, myeloid infiltration, and dynamic changes in adenosine-related metabolites can identify patients most likely to benefit from CD73 blockade.

### 7.3 Spatial and myeloid biomarkers for personalized therapy

Spatial heterogeneity within the TME is a key determinant of immunotherapy response. High adenosine concentrations often co-localize with hypoxic tumor regions and immune-excluded niches, which are enriched with TAMs and MDSCs expressing CD73. These suppressive myeloid populations amplify adenosine-mediated inhibition and prevent effective T-cell infiltration and activation (40, 45). Spatial mapping of CD73 and extracellular adenosine, combined with profiling of immune-cell distribution and phenotype, can provide critical information for personalized therapy. For example, tumors in which CD73-positive TAMs are closely juxtaposed with exhausted T cells may respond preferentially to combinatorial adenosine-axis blockade plus PD-1 inhibitors, whereas tumors with diffuse adenosine production may require dual-targeting strategies, including receptor antagonists or nanoparticle-delivered inhibitors. High-resolution imaging and spatial transcriptomics offer the potential to identify these actionable microenvironments, guiding therapy selection and optimizing combination strategies. From a biomarker perspective, spatial adenosine gradients may explain why bulk immune-cell abundance does not always predict immunotherapy response. A tumor may contain measurable CD8+ T-cell infiltration, but if these cells are spatially restricted to the invasive margin or stromal regions and excluded from CD73-high, hypoxic tumor nests, their antitumor function may remain ineffective (27, 184). Conversely, close spatial proximity between CD73-positive tumor or myeloid cells and exhausted CD8+ T cells may indicate active adenosine-mediated suppression. Therefore, spatial assessment of CD39/CD73 expression, extracellular adenosine distribution, A2AR-positive T cells, A2BR-positive TAM/MDSCs, hypoxia markers, and T-cell localization may provide more informative patient-selection biomarkers than bulk CD73 expression or total CD8+ T-cell density alone.

In summary, CD73 expression, extracellular adenosine levels, and spatially resolved myeloid signatures represent powerful translational tools for guiding cancer immunotherapy. Combining adenosine-targeted strategies with traditional immune checkpoint blockade holds considerable promise for overcoming resistance, enhancing antitumor immunity, and personalizing treatment strategies based on the metabolic and immunologic landscape of individual tumors. These approaches underscore the potential for integrating immune-metabolic biomarkers into routine clinical decision-making, paving the way for more precise, effective, and durable immunotherapeutic interventions (40).

### 7.4 Clinical translation and biomarker challenges

Despite promising clinical progress, several challenges remain for translating adenosine-axis modulation into routine cancer therapy. First, CD73 expression alone may not accurately predict pathway dependence, because adenosine production also depends on extracellular ATP availability, CD39 activity, hypoxia, tissue damage, cellular stress, and the spatial organization of CD73-positive cells. Second, different cellular sources of CD73 may have distinct biological implications. Tumor-cell CD73, stromal CD73, endothelial CD73, myeloid CD73, Treg-associated CD39/CD73, and exosomal CD73 may contribute differently to local immune exclusion, systemic immunosuppression, and treatment resistance (185–187). Third, pharmacodynamic assessment remains underdeveloped. Demonstrating that a CD73 inhibitor reduces enzymatic activity or adenosine accumulation is not equivalent to proving restoration of antitumor immunity. Therefore, future trials should integrate metabolic readouts with immune functional markers, including dendritic-cell activation, CD8+ T-cell infiltration, IFN-γ-associated signatures, granzyme B expression, and reduction of suppressive TAM/MDSC states. The clinical experience with durvalumab plus oleclumab after chemoradiotherapy in unresectable stage III NSCLC illustrates both the promise and complexity of this strategy. The COAST trial supports the feasibility of combining CD73 blockade with PD-L1 inhibition after concurrent chemoradiotherapy, while PACIFIC-9 will further test whether this approach improves outcomes in a larger phase III setting (188, 189). However, these studies also emphasize the need for biomarker-driven patient selection rather than broad, unselected use of adenosine modulators. Ideally, future trials should prospectively evaluate baseline predictive biomarkers, on-treatment pharmacodynamic biomarkers, and resistance biomarkers after progression. This integrated approach may help define whether benefit depends on high CD73 expression, active adenosine generation, myeloid-enriched immune exclusion, chemoradiotherapy-induced purinergic remodeling, or specific combinations of these features.

## 8 Conclusions and future directions

Adenosine signaling has emerged as a central metabolic immune checkpoint in the tumor microenvironment. Through the sequential activity of CD39 and CD73, extracellular ATP released from stressed, dying, or activated cells is converted into adenosine, which subsequently engages A2AR and A2BR to suppress antitumor immunity. Unlike classical immune checkpoints such as PD-1/PD-L1 and CTLA-4, the adenosine axis integrates metabolic stress, hypoxia, tissue damage, and immune-cell dysfunction into a coordinated immunosuppressive program. This makes adenosine signaling a critical link between tumor metabolism and immune escape.

A key insight from recent studies is that adenosine-mediated immune suppression is not restricted to direct inhibition of cytotoxic T cells or NK cells. Instead, it broadly remodels the myeloid compartment of the TME. TAMs and MDSCs can express CD39 and CD73, generate adenosine, and respond to adenosine receptor signaling, thereby creating feed-forward loops that reinforce immune suppression. These myeloid populations suppress T-cell priming, impair effector-cell infiltration, promote T-cell exhaustion, and reduce sensitivity to immune checkpoint blockade. Therefore, myeloid suppression represents one of the most important mechanisms by which the adenosine pathway drives tumor immune escape. Therapeutically, targeting the adenosine axis offers a promising strategy to overcome immunotherapy resistance. Inhibition of CD39 or CD73 can reduce adenosine production at its source, whereas blockade of A2AR and A2BR can prevent adenosine-mediated suppression of lymphoid and myeloid immune cells. These approaches may be particularly effective when combined with PD-1/PD-L1 inhibitors, STING agonists, radiotherapy, photothermal therapy, or adoptive cell-based therapies. Such combinations have the potential to convert immunologically “cold” or adenosine-rich tumors into immune-responsive lesions by restoring cytotoxic T-cell activity, improving antigen presentation, and reducing suppressive myeloid barriers.

Despite these advances, several challenges remain. First, the adenosine pathway is highly context-dependent, and its dominant cellular source may vary across tumor types, disease stages, and treatment conditions. Second, CD39/CD73 expression alone may not fully reflect functional adenosine activity, because extracellular nucleotide availability, hypoxia, receptor distribution, and spatial organization all influence pathway output. Third, the relative contribution of A2AR versus A2BR signaling differs between lymphoid and myeloid compartments, suggesting that patient selection and receptor-specific targeting strategies will be essential for clinical success. Future studies should integrate spatial metabolomics, single-cell transcriptomics, proteomics, and functional immune profiling to define where and how adenosine suppresses antitumor immunity within intact tumors. Spatial mapping of CD39, CD73, extracellular adenosine, A2AR/A2BR expression, TAM/MDSC localization, and exhausted T-cell niches may help identify patients most likely to benefit from adenosine-targeted therapy. In parallel, circulating biomarkers such as serum exosomal CD73 may provide minimally invasive tools for monitoring immunotherapy response and resistance.

Overall, the CD39/CD73–adenosine–A2AR/A2BR axis represents a therapeutically actionable metabolic checkpoint that connects tumor metabolic stress with immune suppression. A deeper understanding of its spatial, cellular, and clinical heterogeneity will be essential for designing rational combination therapies and advancing precision immuno-oncology.
